# Artificial intelligence in medical referrals triage based on Clinical Prioritization Criteria

**DOI:** 10.3389/fdgth.2023.1192975

**Published:** 2023-10-27

**Authors:** Ahmad Abdel-Hafez, Melanie Jones, Maziiar Ebrahimabadi, Cathi Ryan, Steve Graham, Nicola Slee, Bernard Whitfield

**Affiliations:** ^1^ College of Computing & Information Technology, University of Doha for Science and Technology, Doha, Qatar; ^2^ Clinical and Business Intelligence (CBI), eHealth, Queensland Health, Brisbane, QLD, Australia; ^3^ Paediatric Otolaryngology Head and Neck Surgery, Queensland Children’s Hospital, Brisbane, QLD, Australia; ^4^ Medical School, University of Queensland, Brisbane, QLD, Australia; ^5^ Department of Otolaryngology Head and Neck Surgery, Logan Hospital, Meadowbrook, QLD, Australia; ^6^ School of Medicine, Griffith University, Southport, QLD, Australia

**Keywords:** medical referral, electronic health records, clinical decision support system, machine learning, text mining, natural language processing, artificial intelligence

## Abstract

The clinical prioritisation criteria (CPC) are a clinical decision support tool that ensures patients referred for public specialist outpatient services to Queensland Health are assessed according to their clinical urgency. Medical referrals are manually triaged and prioritised into three categories by the associated health service before appointments are booked. We have developed a method using artificial intelligence to automate the process of categorizing medical referrals based on clinical prioritization criteria (CPC) guidelines. Using machine learning techniques, we have created a tool that can assist clinicians in sorting through the substantial number of referrals they receive each year, leading to more efficient use of clinical specialists' time and improved access to healthcare for patients. Our research included analyzing 17,378 ENT referrals from two hospitals in Queensland between 2019 and 2022. Our results show a level of agreement between referral categories and generated predictions of 53.8%.

## 1. Introduction

Every year, millions of patients in Queensland are referred to public outpatient services for continued treatment following consultations in acute care settings. Specialist services may include investigation and diagnosis of conditions not provided by the referring practitioner, and advice and/or provision of treatment and management of complex healthcare conditions. Medical referral letters (referrals) are a form of clinical handover. They should include sufficient information for safe transfer of care and to allow for triaging and categorisation of clinical urgency, prioritisation, and direction of patients to the appropriate specialist outpatient service (1).

**Figure 1:**
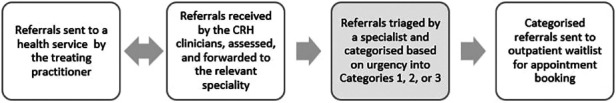
Standard referrals pathway. Third step is where we propose to use the machine learning model. CRH: central referral hub.

Referrals are sent through different lodgment methods to the treating health service including fax, emails, secure transfer system, and General Practitioner Smart Referrals solution (GPSR). Every health service hosts a central referral hub (CRH) to assess referrals and forward them to the relevant specialty or request further information from the referring entity. Figure 1 demonstrates the referral pathways. Once the relevant specialty department receives the referrals, they will triage them and assign a category to them, 1, 2, or 3. Category 1 indicates the highest urgency, where patients must be booked in for an appointment within one month of the received referral date. Categories 2 and 3 represent medium and low urgency, where patients need to be seen within 3 months and one year, respectively (1). The method of receiving referrals is now largely computerised, however, the triaging and categorisation process is manually performed by clinicians, assessing, and reviewing each document in detail. This process is considerably time-consuming.

Queensland health created a decision support tool called the clinical prioritisation criteria (CPC) to help clinicians with triaging and categorisation process (2). CPC are a set of clinical guidelines providing a detailed description of how referrals should be categorised. Several research projects have been completed to measure CPC efficacy. Goh et al. (3) conducted an audit for randomly selected referrals to score them against hospital referrals requirements, they concluded that using CPC is welcomed and needed to improve the quality of referrals. Todd et al. (4) attempted to extract referrals reason by utilising the CPC while using simple similarity calculation method to achieve that. Their presented results show potential in automating referral categorisation. Most recently, Guzman et al. (5) addressed the problem of referrals categorisation and proposed automating the triage of referral letters sent to a spine surgery department using machine learning methods. They used binary labelling for referrals according to urgency. The authors demonstrated the potential for automating the triage of referrals in their work and highlighted the need for further work to solve this problem.

Hospitals and healthcare systems are in the preliminary stages of embracing the capabilities of artificial intelligence (AI) to improve clinical workflow efficiency (6). In our work, we test different methods to categorise referrals received from General Practitioners (GPs) to the ENT specialty in two HHSs (Hospital and Health Services) in Queensland. The proposed categories will be introduced to clinicians as a clinical decision support system (CDSS). This means the decision on triaged referrals categories will be decided by the clinicians. The proposed method is anticipated to enable equitable access for patients needing specialist outpatient services in line with the clinical prioritisation criteria. Moreover, it is expected to reduce the time spent by clinicians for triaging referrals, especially, once the predictions are embedded within the referrals management system. It is important to note potential clinical risks were not analysed in this study and will be looked at in future work.

In a related study (7), the authors introduced a machine learning methodology for medical referral triage using the clinical prioritization criteria (CPC). They collected 3,000 Otorhinolaryngology referrals and used natural language processing (NLP) and cloud services to systematically process and analyse these referrals. The outcomes highlighted the efficacy of this approach demonstrated by a micro F1 score of 0.98. However, the proposed method did not involve the prediction of the CPCs, when the CPC is missing, rather, they progressed with the direct prediction of the category employing similarity functions. Another limitation of their study is that it did not address the imbalance problem within different categories. Instead, they collected 1,000 referrals of each category create a balanced dataset.

## 2. Methods and materials

### 2.1. CPC criteria pre-processing methods

**Figure 2:**
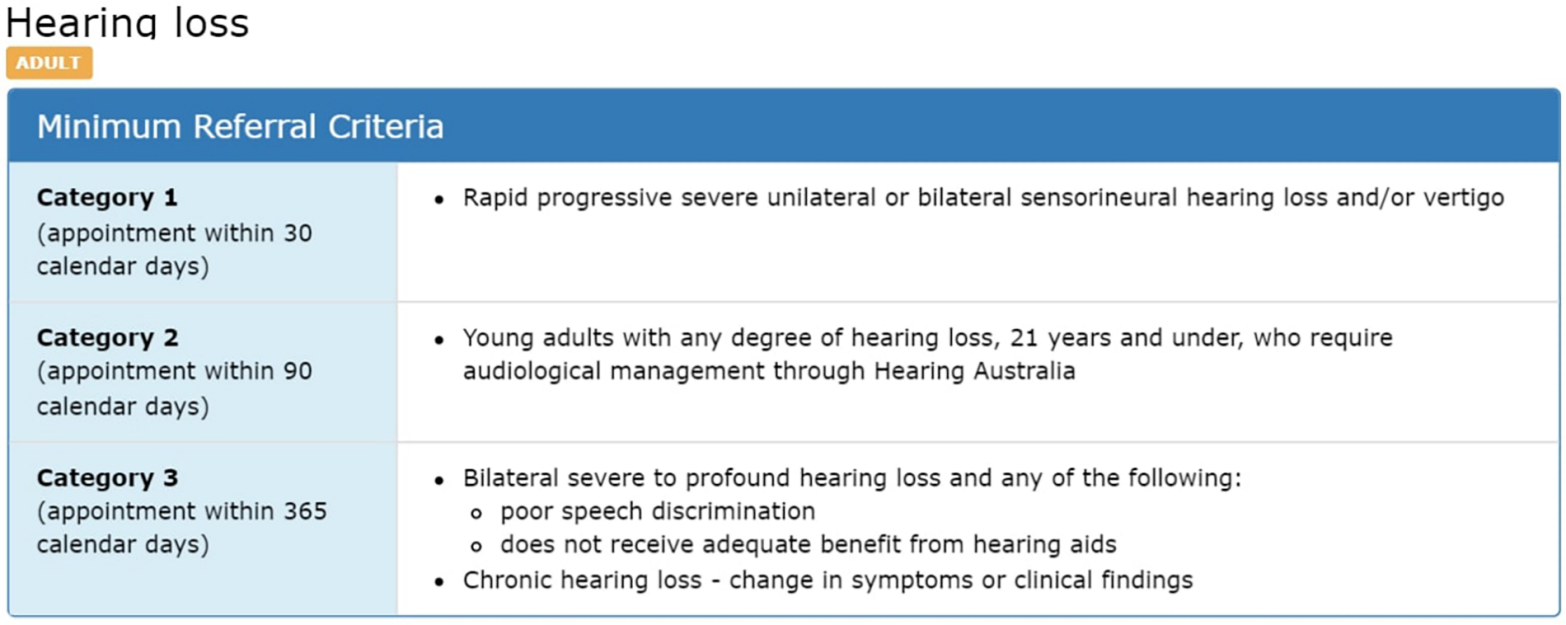
Example of CPC description for “hearing loss” condition (2).

CPC aims to ensure that different public specialist outpatient services around Queensland would apply safe and consistent methods for triaging referrals (8). They also aim to improve the quality of the referrals by applying the minimum required information and to provide a level of consistency for categorisation through different Queensland health services. CRH clinicians may return the referrals to the requesting practitioner if they do not meet the CPC guideline standards (3). An example of how the CPC guidelines is structured for adult hearing loss is illustrated in Figure 2. It demonstrates the criteria for classifying hearing loss referrals for adults as category 1, 2, or 3.

**Figure 3:**
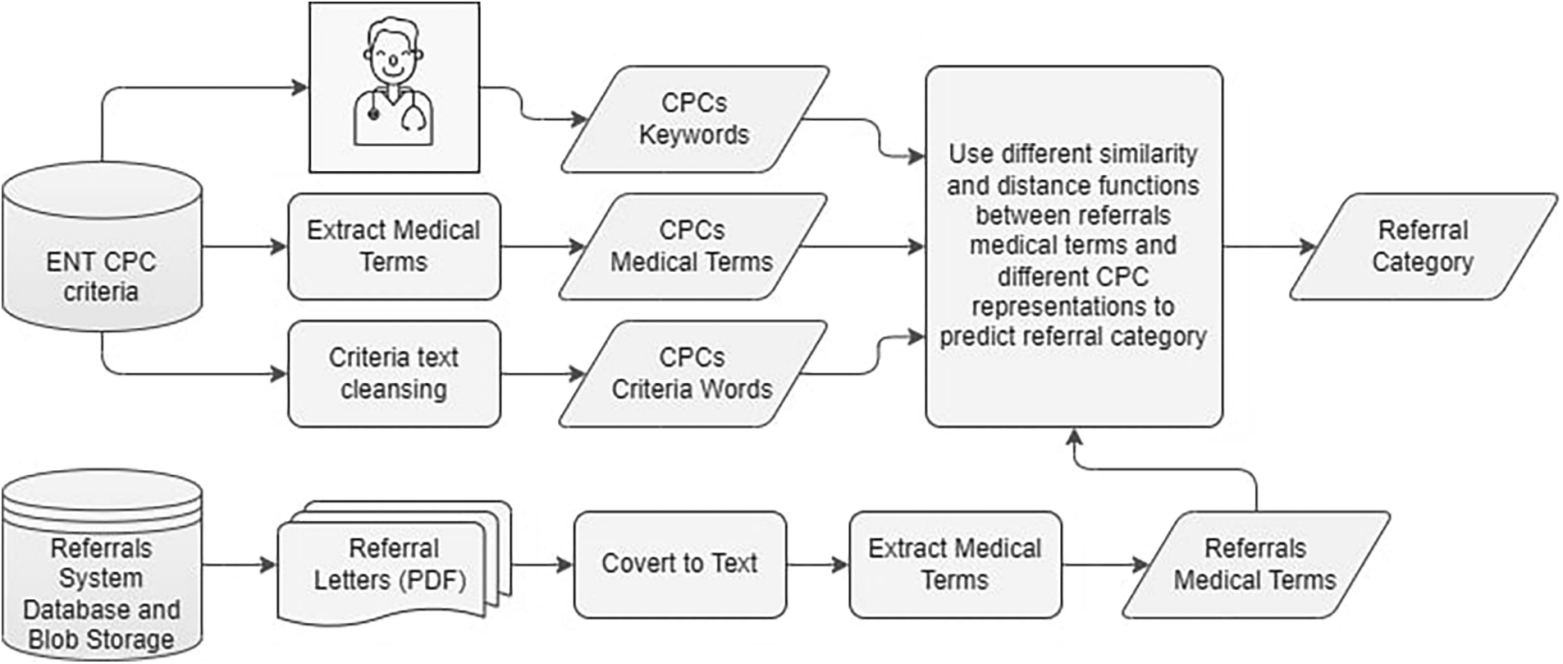
Data collection and preparation process.

The CPC criteria preprocessing are completed for the three categories and used as combined unit for the CPC prediction step, or as separate units for the category prediction process. The CPC criteria are preprocessed in three separate ways as shown in Figure 3. Like the referrals, we process CPC using Amazon Comprehend Medical© tool (9) to extract medical terms. We keep this method consistent with the referrals medical terms processing to make sure we do not impact the similarity between them. The second method involves applying text cleansing and preprocessing directly to the CPC criteria text. Again, this process is consistent with the text cleaning conducted for the medical terms step. Finally, we consult with clinicians to identify unique keywords representing each category within each CPC criteria. We test the three different representations of the CPC in our method.

### 2.2. Data collection and preparation process

We collected three years (2019–2022) referrals for ENT specialty from two different HHSs, 17,378 referrals in total. 5,688 referrals are paediatric, while 11,690 referrals are adult. The collected data include attachment files associated with referrals. Figure 3 shows the data collection and preparation process, as we perform preprocessing on referrals portable document format (PDF) files and CPC criteria text (2). We identify PDF files representing the referrals main letters, and then we convert them into text using Azure Cognitive Services© (Document Intelligence v3.1) (10). We extract the relevant medical terms used in the referrals using Amazon Comprehend Medical© tool (9). This tool identifies relevant medical entities in text and generates JavaScript Object Notation (JSON) files for each discovered entity containing detailed information about it. For each medical entity, the tool assigns a score between 0 and 1 which indicates the tool's confidence of the detected information. We include entities with a larger score or equal to 0.5. Table 1 shows the entities we collected from the JSON files as medical terms, which were selected after careful consideration from the business analysts and clinicians involved in this project (11). Collected entities include signs, symptoms, and diagnosis for present or historical illness, medications using both generic and brand names, procedures, and anatomy.

**Table 1:** Entities collected from JSON files generated by Amazon comprehend medical© tool (11).

Category	Types
Medical_Condition: signs, symptoms, and diagnosis of medical conditions.	DX_Name: includes present illness, reason for visit, and medical history
Medication: medication and dosage information for the patient	1- Generic_Name: The non-brand name, ingredient name, or formula mixture of the medication or therapeutic agent. 2- Brand_Name: The copyrighted brand name of the medication or therapeutic agent
Test_Treatment_Procedure: detects the procedures that are used to determine a medical condition.	1- Treatment_Name: Interventions performed over a span of time for combating a disease or disorder. This includes groupings of medications, such as antivirals and vaccinations. 2- Procedure_Name: Interventions as a one-time action performed on the patient to treat a medical condition or to provide patient care.
Anatomy: references to the parts of the body or body systems and the locations of those parts or systems	Direction: Directional terms.

Named Entity Recognition (NER) is a fundamental component of natural language processing (NLP) that focuses on identifying and extracting specific named entities from text. Amazon Comprehend Medical© (9) use NER to identify medical entities within unstructured medical text. In NER, the goal is to maximize the conditional probability distribution over tags given an input sequence. The model consists of a character encoder, word encoder, and decoder/tagger (11). The word encoder uses bidirectional Long Short-Term Memory (BiLSTM) (12) to encode word-level representations. The decoder uses the concatenated output of the word encoder along with label embeddings as input to generate predictions. Additionally, the decoder model incorporates entity extraction predictions to provide more context for trait detection. The proposed architecture enhances predictions, such as negation, based on the entity prediction distribution (11).

Once the medical terms are extracted, we clean them by removing special characters, numbers, punctuation, and stop words. Then, we perform word stemming to be able to group similar medical terms as one feature in the ML (Machine Learning) model. We utilised Python libraries for the preprocessing step including gensim (version 3.8.3), and nltk (version 3.8.1) (13, 14).

### 2.3. CPC prediction methods

**Figure 4:**
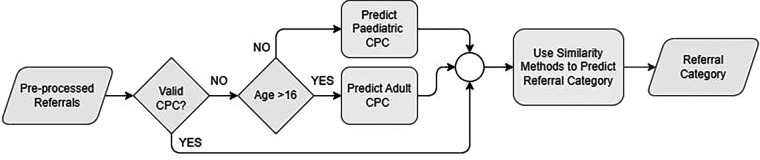
CPC prediction flowchart.

Referrals are expected to be associated with specific CPC, assigned by GPs using the GPSR system. This system allows GPs to create and submit electronic referrals while providing structured templates to improve the quality of the submitted referrals. One field introduced through the GPSR system is the CPC. However, since only referrals submitted through GPSR have CPC specified, and because not all GPs select a CPC for the referral before sending it, we have only 9.6% of the total referrals (17,378) with CPC, Table 2 provides more details. The availability of CPC for referrals is a key factor in our method of predicting the category. To be able to categorise referrals without CPC, first, we propose to predict the CPC and assign it to the referral. Figure 4 shows the flowchart of the proposed method for the referral's categorisation process. First, we check if the referral has a CPC attached to it, and check if this CPC value matches one of the identified CPC lists (2). If not, then we proceed to predict CPC for that referral using the proper age group CPC based on patient's age.

**Table 2:** ENT referrals data description by facility and submission method.

	All referrals			Referrals with CPC		
Referrals	SCHHS	TVHHS	Total	SCHHS	TVHHS	Total
All referrals	10,822	6,556	17,378	1,082	607	1,689
GPSR	2,557	1,025	3,582	1,072	607	1,679
Email	3,469	2,516	5,985	8	0	8
Fax	4,725	2,882	7,607	2	0	2
Manual entry	68	132	200	0	0	0
STS	3	1	4	0	0	0

GPSR, general practitioner smart referrals; STS, secure transfer system; SCHHS, Sunshine Coast hospital and health services; TVHHS, Townsville hospital and health services.

**Figure 5:**
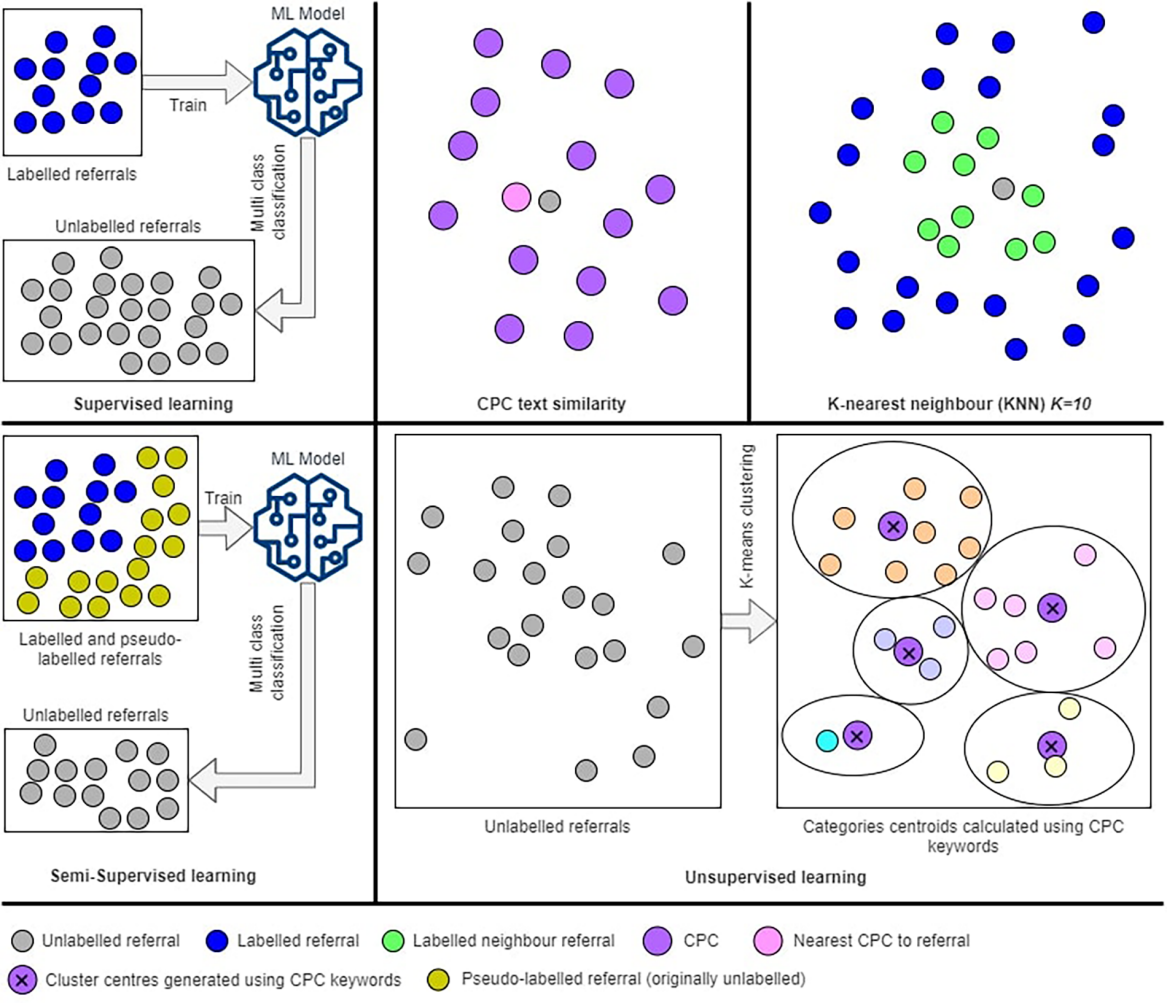
Graphical illustration of the CPC prediction proposed methods.

CPC are defined as per age groups into adult and paediatric CPC, across all outpatient specialty service. Since we have a pool of referrals, not assigned to an age group, we follow clinical workflow and recommendation to use the age of 16 as a cutoff to identify referral age group. We calculate a patient's age at the referral's submission date. Once we identified the age group, we performed a CPC prediction method. Figure 5 illustrates all the proposed methods for CPC prediction.

According to the data presented in Table 2, we possess 1,689 labeled referrals with Clinical Procedure Codes (CPC), which we utilize to predict CPC for other referrals lacking labels (unlabeled). The task of predicting CPC involves multiclassification, where we have 20 CPCs for adult referrals and 13 CPCs for pediatric referrals in the field of ENT. Table 3 shows the CPC and frequencies of their occurrences on the labeled dataset. We can see that some CPC have 0 labels, such as “neck mass”. One solution to overcome this problem is to collect and process larger datasets, which is not feasible for us in this project. Since our aim is to predict the category (not the CPC) and predicting CPC was considered a necessity to achieve our goal, we decided to measure the performance of the introduced methods (predicting CPC) through the categorisation accuracy. If a specific method for predicting CPC is producing better categorisation accuracy, then we recommend that method.

**Table 3:** List of CPC and the frequency of occurrence in the labeled dataset.

ENT CPC	CPC condition description	CPC condition frequencies in the labeled referrals
Adult CPC	Allergic rhinitis/nasal congestion/obstruction	0
	chronic ear disease	82
	dizziness/vertigo	37
	dysphagia	103
	dysphonia	124
	ear drum perforation	20
	epistaxis (recurrent)	31
	facial nerve palsy	2
	head and neck mass	100
	hearing loss	180
	nasal fracture (acute)	28
	obstructive sleep apnoea	42
	oropharyngeal lesions	47
	primary parathyroid adenoma	3
	rhinosinusitis (chronic/recurrent)	214
	salivary tumour	13
	sialolithiasis (salivary stones)	10
	thyroid mass	27
	tinnitus	45
	tonsillitis (recurrent) or tonsillar enlargement	127
Paediatric CPC	dysphonia/hoarseness	4
	epistaxis (recurrent)	5
	hearing loss	24
	Nasal allergic rhinitis/congestion/obstruction	0
	Nasal fracture (acute)	0
	Neck mass	0
	otitis externa	3
	Otitis media—acute otitis media with or without perforation (AOMwiP/AOMwoP)	0
	Otitis media—with effusion (OME or glue ear)	0
	perforated eardrum/chronic suppurative otitis media (CSOM)	5
	sleep disordered breathing/obstructive sleep apnoea	36
	stridor	3
	tonsillitis (recurrent)	50

ENT, ear nose and throat; CPC, clinical prioritisation criteria.

#### 2.3.1. Text similarity

In this method, we use the CPC description to predict CPC for referrals by finding the closest CPC description to the referral text. This method is a variation of content-based recommender systems, as it does not depend on the labelled dataset (15). There are many different methods available to measure how close two texts are statistically or semantically, with respect to their use of words or characters (16). We tested selected methods covering traditional distance-based text similarity methods, namely, Cosine similarity, and Euclidean distance (17), and representation-based approaches including phrase-based, Jaccard similarity (18), and character-based, Levenshtein distance (19). The outcomes of the four similarity methods are combined using weighted average method to calculate the closest CPC to the referral text. Weights are represented by the accuracy of the similarity methods on predicting referral categories using referrals with assigned CPC.

All the used similarity methods are based on statistics and allow comparison of two different length strings. The Cosine and Euclidean methods transfer text to vector of words frequencies in the text. The Jaccard method is more basic as it only finds the ratio of shared words to the total number of words in both documents. Levenshtein distance is an edit distance, which is calculated by finding the number of edits required to get text (a) to become equal to text (b). The higher the number of edits, the greater the distance between two texts. This function is defined in the equation in a recursive method for simplicity, however, it can be implemented using a matrix.

$$\mathrm{Cos}(A,B)=\frac{\sum_{i=1}^{n}A_{i}B_{i}}{\sqrt{\sum_{i=1}^{n}A_{i}^{2}}\sqrt{\sum_{i=1}^{n}B_{i}^{2}}}$$

$$\mathrm{Euc}(A,B)=\sqrt{\overset{n}{\underset{i=1}{\sum }}{\left(A_{i}-B_{i}\right)}^{2}}$$

$$\mathrm{Jac}(A,B)=\frac{\left|A\cap B\right|}{\left|A\cup B\right|}$$

$$\mathrm{Le}v_{A,B}(i,j)=\{\begin{array}{c} \begin{array}{cc} \;\max(i,j)\; & \;\mathrm{if}\;\min(i,j)\;=\;0, \end{array}\\ \begin{array}{cc} \min\{\begin{array}{c} \;\mathrm{Le}v_{A,B}(i-1,j)+1\;\\ \;\mathrm{Le}v_{A,B}(i,j-1)+1\;\\ \mathrm{Le}v_{A,B}(i-1,j-1)+1+1_{\left(A_{i}\neq B_{i}\right)} \end{array} & \;\mathrm{otherwise} \end{array}. \end{array}$$

$$\mathrm{CPC}(A)=\underset{B\in \mathrm{CPCs}}{\mathrm{argmax}}\left(w_{1}\times \mathrm{Cos}(A,B)-w_{2}\times \mathrm{Euc}(A,B)+w_{3}\times \mathrm{Jac}(A,B)-w_{4}\times \frac{\mathrm{Le}v_{A,B}(i,j)}{\left(l(A)+l(B)\right)}\right)$$

*A* and *B* represent the two texts compared. In the Cos and Euc equations, *n* represents the number of elements (words) in the vector space. In the Lev equation, *i* and *j* are temporary variables used for recursive calculation of the Levenshtein distance, where they are initiated to *A*, and *B*, respectively. In the CPC(A) equation, we find the final CPC for the referral *A*, *w* represents the weight assigned to each similarity method, *l* is length of the text in each referral, and the argmax operation will return the CPC which will generate the maximum value. Using the weighted average method, in the CPC(A) equation above, we calculate the similarity between referral *A* and the set of CPC text *B,* where B∈CPCs. The CPC associated with the text that returns the highest similarity will be the CPC assigned to the referral *A*. Notice that we add similarity metrics Cos and Jac, and we deduct distance metrics Euc and Lev.

We divide the Levenshtein distance by the maximum possible distance between two strings of given lengths to normalize the value int the range from 0 to 1, to be compatible with other similarity methods. Notice that we subtract Euc and Lev because they represent distance not similarity.

#### 2.3.2. *K*-nearest neighbour (KNN)

The KNN method measures similarities between referrals, for each unlabeled referral we calculate similarities with all labeled referrals and generate a list of 10 referrals with highest similarity larger than 0 (15, 20). For the similarity calculation process, we use Euclidean distance converted to similarity as per the equation below. Finally, we find the closest CPC of the identified neighbours to assign it to the unlabeled referral by calculating CPC weights using the similarity scores.

$$\mathrm{Euc}\_\mathrm{sim}(A,B)=\frac{1}{e^{-\mathrm{Euc}(A,B)}}$$

$$\mathrm{CPC}(A)=\underset{\mathrm{CPC}\in N_{\mathrm{CPCs}}}{\mathrm{argmax}}\left(\underset{R\in N,R_{\mathrm{CPC}}=\mathrm{CPC}}{\sum }\mathrm{Euc}\_\mathrm{sim}(A,R)\right)$$

*N* represents the set of identified neighbours, *N*_CPC_ is the set of CPC appears within the neighbours’ referrals, *R* is a neighbour referral, and *R*_CPC_ is the CPC assigned to the neighbour referral.

#### 2.3.3. Supervised learning methods

Supervised learning approach uses labeled data to train a classification model which can be used to predict CPC for the unlabeled referrals. Our problem is a multiclass classification, where each referral can be assigned only on CPC, and we have more than two CPC to select from. Initially, we utilized labeled referrals to perform a stratified 5-fold cross-validation classification experiment, with 80% of the data allocated as the learning dataset and 20% as the testing dataset. We incorporated a total of 1,689 labeled referrals in this process. Within each fold, 338 referrals (20%) were set aside for testing the model, which was trained using the remaining referrals. By employing stratified splitting, we ensured that the distribution of outcome values was similar in each fold. Upon completing the 5-folds, we had utilized every record in the dataset for testing exactly once. Once we optimize the ML model to the best set of hyperparameter values that achieve best accuracy scores, we use the model to predict CPC for the unlabeled data.

For feature extraction we use CountVectorizer function from scikit learn feature extraction library on medical terms extracted from referrals (21). This function will generate a matrix with counts for medical terms. We include unigrams and bigrams features with minimum 30 occurrences on different referrals if they do not appear in more than 80% of the referrals. These parameters aim to remove noise from the data, such as rare/common medical terms.

Several machine learning methods were tested to generate a multiclass machine learning model to predict CPC. We tested three multiclass strategies using linear SVM (support vector machine) method (22), including one vs. rest (OvR), one vs. one (OvO), and error-correcting output-code multiclass strategy (23). These strategies provide details on how multiple binary classifiers are combined to build the multiclass classifier. The OvR strategy will build one binary class for each class (in this case CPC) predicting if a referral belongs to this CPC or to any other CPC. The OvO method, however, builds a binary classifier between every unique pair of CPC, generating in total n(n−1)/2 classifiers, where *n* is the total number of CPC (24). The error-correcting method on the other hand, represents each class (CPC) with a unique binary code, and builds a binary classifier per bit (23). Moreover, we tested different methods by fitting a stochastic gradient descent (SGD) optimisation (25) method including logistic regression, linear SVM (22), and perceptron (26). Other methods tested as well include random forest (27), gradient boosting (28), multilayer perceptron (29), light gradient boosting machine (light GBM) (30), extreme gradient boosting (Xgboost) (31). Grid search hyperparameter tuning were conducted for each of the implemented methods (hyperparameters values for the implemented methods are provided in Supplementary file S2).

#### 2.3.4. Semi-supervised learning

This method helps with addressing the problem of having insufficient labelled data to build an accurate ML model using supervised learning. In our dataset, we have only 9.7% of labelled data, hence, using semi-supervised learning method could improve the accuracy by increasing the size of the training dataset (labelled dataset). This method allows supervised algorithms to learn from unlabeled data. The self-learning algorithm (32) is an iterative prediction process which assigns pseudo-labels for the unlabeled data and adds them to the training set. The decision of adding a pseudo-label is made using a ML algorithm with a high cut-off threshold, we use 0.75, rather than the normal binary prediction threshold of 0.5. The classifier will iterate until the specified maximum iteration is reached, or no pseudo-labels were added to the training set in the previous iteration. Figure 5 shows an illustration of the semi-supervised learning process.

#### 2.3.5. Unsupervised learning using *K*-means clustering

Unsupervised learning is applied to unlabeled data; hence, we ignore the labels (CPC) assigned to the 9.7% of the dataset and we treat them as unlabeled. The *k*-means method (33) will divide referrals into *k* clusters. This algorithm requires the number of clusters k to be identified. In our method, we know exactly the value of *k*, where each cluster represents exactly one CPC. We have 20 CPC for adults (*k* = 20) and 13 CPC for paediatric (*k* = 13). This indicates that we must build two separate k-means models, one for adult referrals and one for paediatric referrals. The algorithm aims to minimize the within-cluster sum of squares of the distances between data and centroid.

$$\overset{n}{\underset{i=1}{\sum }}\underset{\mu \in C}{\min}(x_{i}-\mu^{2})$$

The *k*-means algorithm starts by initializing a centroid value for each cluster *C*, indicated as *µ*. In each iteration, we assign referrals to the closest cluster using Euclidean distance between referrals and clusters' centroids. Centroids are updated with new data from added referrals after each iteration. The algorithm converges once the centroids are stable and stop changing in the next iterations. Since we have sufficient information about every CPC (cluster), we generate a list of seeds from the CPC medical terms and keywords to initialize every cluster centroid. This will ensure that every cluster represents one CPC in which we used its medical terms and keywords.

### 2.4. Predicting referral categories

The categorisation process completed on the collected historical referrals was not fully compliant with the CPC criteria. Hence, we do not have a ground truth to measure the proposed model accuracy. However, we use the assigned category to the referrals as a guide to compare the different methods we implemented, we call this metric “level of agreement.” Figure 4 shows the confusion matrices for the best performing method, which was the Levenshtein distance between 1-gram CPC keywords (produced by clinicians) and referrals medical terms.

From the results we notice the increase in the level of agreement when we used GPSR referrals only and GPSR referrals with CPC assigned to them. When we compare the predicted categories in comparison to the assigned categories, we notice that our method produces results that are more consistent with the CPC criteria. For example, the “head and neck mass (ENT)” CPC criteria indicates that all referrals with this CPC must be categorised as category 1. While our method categorised all the 101 referrals in the adult dataset as category 1, the assigned categories show 22 referrals were triaged as category 2, and 2 referrals as category 3. This pattern is repeated over most CPC. This indicates that patients with similar conditions were prioritised differently. Our method does not suffer this drawback. It can provide more equitable access to specialist outpatient services for different patients from various locations around the state.

## 3. Results

The data set used for this method includes historical ENT referrals, which were categorised by clinicians in each hospital. Since the introduction of CPC, clinicians are urged to use them for categorisation. However, the categorisation process was not always consistent with the CPC categorisation guide. This is due to varied reasons such as referrals quality, availability of resources, clinical judgment subjectivity, comorbidities, and other reasons. As we use the CPC to perform this method, the ideal outcome would be re-labeling the data to be aligned with the CPC criteria. However, this option is very costly as it requires the categorisation of 17,378 referrals. We decided to utilise the current categories on the assumption that they will be in line with the CPC criteria. We measured the level of agreement between the predicted categories and the actual assigned categories to the referrals to measure the best performing method. The higher the level of agreement the better the performance of the proposed method, even though this is not a reflection of the method's accuracy.

Table 4 shows the results for the proposed methods to predict CPC across the four similarity methods to predict the categories. The best performing method was using text similarity to predict CPC with using Levenshtein distance between referral medical terms and CPC keywords identified by clinicians with a level of agreement with historical categorisation of 0.538. Note that random categorisation is assumed to achieve a 0.333 level of agreement as we have a ternary classification.

**Table 4:** Level of agreement of the proposed methods with the historical categories.

	Similarity method	Cosine similarity			Euclidean distance			Jaccard similarity			Levenshtein distance		
	CPC pre-processing method	CPC criteria words	CPC medical terms	CPC Keywords	CPC criteria words	CPC medical terms	CPC Keywords	CPC criteria words	CPC medical terms	CPC Keywords	CPC criteria words	CPC medical terms	CPC Keywords
Text similarity	–	0.474	0.506	0.501	0.482	0.514	0.510	0.399	0.441	0.424	0.530	0.528	**0** **.** **538**
KNN	–	0.196	0.196	0.198	0.203	0.203	0.199	0.181	0.196	0.201	0.457	0.471	0.485
Supervised learning	Logistic regression	0.336	0.350	0.354	0.344	0.354	0.361	0.217	0.325	0.322	0.433	0.372	0.395
	LinearSVM (OvR)	0.328	0.343	0.339	0.336	0.346	0.356	0.210	0.338	0.368	0.447	0.366	0.376
	LinearSVM (OvO)	0.316	0.330	0.331	0.325	0.330	0.351	0.202	0.338	0.331	0.428	0.347	0.385
	LinearSVM (error-correcting)	0.330	0.342	0.339	0.338	0.345	0.349	0.206	0.344	0.351	0.452	0.364	0.398
	SGD Perceptron	0.348	0.354	0.361	0.355	0.358	0.361	0.223	0.341	0.339	0.435	0.355	0.394
	Random forest	0.334	0.348	0.349	0.342	0.352	0.353	0.217	0.356	0.358	0.449	0.352	0.361
	Gradient boosting	0.338	0.352	0.361	0.344	0.356	0.374	0.216	0.335	0.336	0.424	0.361	0.374
	MLP	0.251	0.241	0.241	0.259	0.245	0.255	0.131	0.282	0.284	0.359	0.263	0.274
	LGBM	0.336	0.351	0.364	0.342	0.352	0.369	0.230	0.352	0.353	0.425	0.350	0.351
	Xgboost	0.339	0.356	0.359	0.346	0.359	0.361	0.210	0.349	0.351	0.442	0.368	0.367
Semi-supervised learning	Logistic regression	0.343	0.360	0.365	0.350	0.364	0.370	0.238	0.345	0.352	0.432	0.365	0.389
Unsupervised learning	K-means	0.439	0.442	0.445	0.443	0.446	0.448	0.369	0.456	0.461	0.494	0.491	0.501

CPC, clinical prioritisation criteria; KNN, k nearest neighbours; SVM, support vector machine; OvR, one vs. rest; OvO, one vs. one; SGD, stochastic gradient boosting; MLP, multilayer perceptron; LGBM, light gradient boosting machine; Xgboost, extreme gradient boosting.

Bold values indicate the best performing methods.

**Figure 6:**
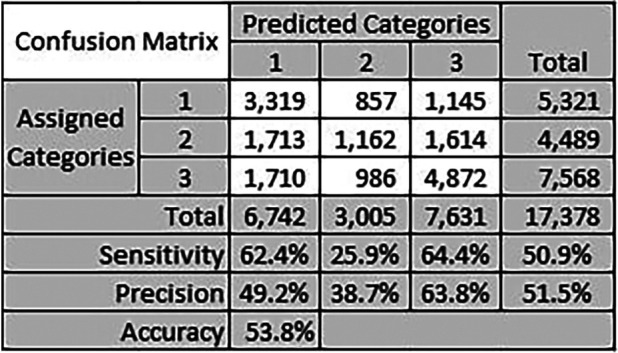
Confusion matrix for the text similarity method using Levenshtein distance between referral medical terms and CPC keywords identified by clinicians.

Figure 6 shows a confusion matrix for the best performing method. The confusion matrix shows the performance of the proposed method at each category level, and the overall performance as well. We calculate the precision and sensitivity per category, where the overall metrics values are simply the average of all values over all categories. Precision is a measure of true positive TP predictions to the overall positive predictions, positive and negative FP. Sensitivity, on the other hand, measures the true positive TP predictions to the overall positive values in the dataset, also known as recall. We also calculate accuracy which computes the true positive predictions to the total number of predictions.

The best performing category was category 3 with precision of 63.8% and sensitivity of 64.4%. We believe the reason is because this is a majority class, where the total number of historical referrals for category 3 is 7,568. Category 1 sensitivity was also close to category 3 sensitivity with 62.4%. These results do not reflect the actual accuracy of the CPC categorisation proposed method, but they reflect the expected trends with similar datasets. The number of referrals per category impacts the precision and sensitivity of categorisation for that category.

## 4. Discussion

### 4.1. CPC pre-processing methods

In our study, we explored three different approaches to preprocess the CPC criteria text, aiming to identify the most effective method regardless of other factors such as the CPC prediction method or category prediction process. Analyzing the results presented in Table 4, we consistently observed that both the CPC medical terms and CPC keywords methods outperformed the CPC criteria words method across various techniques. This outcome was as expected since the CPC criteria text could contain some noise following the text cleansing process.

An interesting finding emerged when comparing the performance of keywords identified by our clinicians (provided as a Supplementary file) with the medical terms extracted by AWS Comprehend Medical. The clinician-derived keywords exhibited a slightly better performance compared to the medical terms extracted by AWS Comprehend Medical. Specifically, the level of agreement for the CPC keywords method was measured at 0.538, whereas the CPC medical terms method achieved a level of agreement of 0.528. This indicates that the CPC keywords method showed a marginal improvement of 1.89% over the CPC medical terms method. To determine the statistical significance of this improvement, we calculated the *p*-value using a t-test within a 95% confidence interval. A *t*-test is a statistical test used to compare the means of two groups and determine whether the difference between them is statistically significant. The resulting *p*-value of the *t*-test was found to be p=.538,p>.05, indicating that the observed improvement was not statistically significant. Therefore, we cannot confidently conclude that the difference in performance between the CPC keywords and CPC medical terms methods is significant. Based on these findings, our study suggests that utilizing the AWS Comprehend Medical tool alone is sufficient for extracting medical terms, eliminating the need for manual extraction by specialists. Despite the slight advantage demonstrated by the clinician-derived keywords, this advantage was not statistically significant. Therefore, the automated extraction process provided by AWS Comprehend Medical proves to be a reliable alternative, offering comparable performance without requiring manual specialist involvement.

### 4.2. CPC prediction methods

In section 2.3, we discussed that CPC prediction is currently only conducted for referrals without CPC specified by the referring GP, which accounts for approximately 90% of the dataset. However, we anticipate this percentage to decrease in the future as Queensland Health actively provides trainings to GPs on utilizing the new GPSR system and emphasizes the importance of providing comprehensive information and details when submitting referrals to outpatient services. Improving referral quality and capturing relevant details remains a significant challenge in this task. Hence, we anticipate the performance of our method to improve over time as the referral's quality improve. A previous study in this area focused on predicting categories using similarity functions, without predicting CPCs (7).

One of the limitations of the proposed unsupervised learning method is it relies on a predetermined number of clusters. We had to fix this number to align with the CPCs we intended to predict (2). Consequently, the clusters are not determined by the data, impacting the performance of the clustering method. Another significant limitation is the small dataset size and the under-representation of some of the CPCs in the dataset, which can undermine the confidence and reliability of the outcomes obtained, as it may lead to skewed or biased results, hindering the model's ability to generalize effectively to all possible outcomes.

Regarding our results, we found that the text similarity method exhibited superior performance compared to all other methods, with unsupervised learning ranking second. In general, supervised learning algorithms are expected to excel when sufficient data is available for model training, allowing them to learn underlying relationships and patterns necessary for accurate class label prediction. However, due to the limited amount of data available to accurately predict CPC labels in our study, we propose employing the text similarity method as it demonstrated the highest performance among the tested approaches.

### 4.3. Predicting referral categories

In a separate research investigation, researchers employed Cosine similarity to generate a similarity score for two medical documents (34). The authors proposed the utilization of text pre-processing techniques, such as removing punctuation, converting text to lowercase, tokenization, stop word removal, and stemming. They represented the pre-processed keywords using term frequency and calculated weighted Cosine similarity based on the counts. In our study, we followed all the pre-processing steps outlined in (34), and additionally incorporated Named Entity Recognition (NER) to diminish any noise within the extracted text from the referral documents.

In our work, three of the four similarity and distance methods used to predict referral categories produced comparable results, namely the cosine similarity, Euclidean distance, and Jaccard similarity. However, the fourth method, Levenshtein distance, was significantly better than other methods across all the conducted experiments. Levenshtein distance is preferred for measuring the similarity between strings as it considers the operations needed to change one string to another, making it suitable for comparing strings with different lengths or containing typos. In the referrals extracted medical terms we notice several terms used to describe same symptom, for example, the words “blocked”, “blockage”, “block”, “blockednose” etc. We try to reduce words to the smallest shape, but it does not work all the times, especially if there was a typo. Hence, the Levenshtein distance produced the best results in our experiment.

In the context of other disease groups, it is imperative to acknowledge that the predictive models are anticipated to exhibit dissimilarities and, therefore, necessitate separate and rigorous testing procedures. It is crucial to emphasize that the model developed and tested in this study pertains specifically to the ENT specialty, and thus, the results obtained cannot be generalized to other medical specialties without the validation and adaptation of the model to each distinct domain. However, it is worth noting that the methodology proposed in this study can be readily replicated and applied to other medical specialties with appropriate modifications and validations to ensure its suitability and efficacy in those specific contexts.

## 5. Conclusion

In this research, we present a solution for automating the categorization of medical referrals for ear, nose, and throat (ENT) specialists using clinical prioritization criteria (CPC) guidelines. Through our experiments, we discovered that the Levenshtein distance was a more effective measure of similarity between strings than other distance and similarity methods. Additionally, our findings indicate that using the AWS Comprehend Medical tool for pre-processing CPC can produce results that are as accurate as manual keyword extraction by healthcare specialists.

Finally, our results indicate that the text similarity method and unsupervised learning with predetermined seeds are the best methods for predicting CPC for medical referrals. One significant limitation is the small dataset size and the under-representation of some of the CPCs in the dataset, which can undermine the confidence and reliability of the outcomes obtained. In conclusion, our proposed method exhibits promising potential to support clinicians efficiently categorizing medical referrals based on clinical urgency. In future, further development and testing in the context of clinical risks must be conducted before using this tool in a clinical context as a decision support tool. Large language models (LLM) also may provide an alternative solution for the problem described in this work. LLM models can be explored in future, tested, and compared with the proposed methods in this work.

## Data Availability

The datasets presented in this article are not readily available because of reasonable privacy and security concerns and requirements imposed by Queensland health relevant ethics committees. Requests to access the datasets should be directed to ahmad.abdel-hafez@health.qld.gov.au.
