# Supplementary material for: Artificial intelligence in medical referrals triage based on Clinical Prioritization Criteria
Source: Front Digit Health. 2023 Oct 27;5:1192975. doi: 10.3389/fdgth.2023.1192975 (PMC10642163; doi:10.3389/fdgth.2023.1192975)
Supplement: Supplementary file 1 [file Datasheet1.pdf]

Supplementary File 1:

Equations:

$$Precision_i = \frac{TP_i}{TP_i + FP_i}$$

$$Sensitivity_i = \frac{TP_i}{TP_i + FN_i}$$

$$Accuracy = \frac{TP_1 + TP_2 + TP_3}{Total}$$

Results:

Confusion Matrix:

Confusion Matrix		Predicted Categories			Total
		1	2	3	
Assigned Categories	1	3,319	857	1,145	5,321
	2	1,713	1,162	1,614	4,489
	3	1,710	986	4,872	7,568
	Total	6,742	3,005	7,631	17,378
	Sensitivity	62.4%	25.9%	64.4%	50.9%
	Precision	49.2%	38.7%	63.8%	51.5%
	Accuracy	53.8%			

Figure S1-1: Confusion matrix for text similarity method using Levenshtein distance with CPC Keywords

Code:

```
from math import sqrt, pow, exp
def squared_sum(x):
    """ return 3 rounded square rooted value """
    return round(sqrt(sum([int(a)*int(a) for a in x])),3)

def jaccard_similarity(x,y):
    intersection_cardinality = len(set.intersection(*[set(x), set(y)]))
    union_cardinality = len(set.union(*[set(x), set(y)]))
    return intersection_cardinality/float(union_cardinality)

def cos_similarity(X,Y):
    rvector=[]; x =[];y =[]
    rvector = X.union(Y)
    for w in rvector:
        if w in X: x.append(1)
        else: x.append(0)
        if w in Y: y.append(1)
        else: y.append(0)
    numerator = sum(int(a)*int(b) for a,b in zip(x,y))
    denominator = squared_sum(x)*squared_sum(y)
    return round(numerator/float(denominator),3) if denominator !=0 else 0

def euclidean_distance_to_similarity(X,Y):
    rvector=[]; x =[];y =[]
    # form a set containing keywords of both strings
    rvector = X.union(Y)
    for w in rvector:
        if w in X: x.append(1) # create a vector
        else: x.append(0)
        if w in Y: y.append(1)
        else: y.append(0)
    distance = sqrt(sum(pow(int(a)-int(b),2) for a, b in zip(x, y)))
    return 1/exp(distance)

import string
def remove_punctuation(s):
```

```python
    s=s.replace(',','')
    return s

def prepare_entities_df(df,e):
    l_entities_w = prepare_entities_df_Words(df,e)
    l_entities = []
    s = set()
    print(df.shape[0])
    for i in range(df.shape[0]):
        for each in df[e][i].split(','):
            s.add(remove_punctuation(each).lstrip(" ").strip("]['"))
        for w in l_entities_w[i]:
            s.add(w)
        l_entities.append(s.copy())
        s.clear()
    return l_entities

import re
def prepare_entities_df_Words(df,e):
    l_entities = []
    s = set()
    print(df.shape[0])
    for i in range(0,df.shape[0]):
        for each in re.split(', | |,',df[e][i]):
            each = remove_punctuation(each).lstrip(" ").strip("]['")
            each = remove_stopwords(each)
            each = each.replace('\ ','').lower()
            if each != '':
                s.add(each)
        l_entities.append(s.copy())
        s.clear()
    return l_entities



s_MT_Set_W = set()
s_MT_Set_PH = set()

def prepare_MT(MT):
    sw = prepare_MT_words(MT)
    s = set()
    for each in MT.split(','):
        s.add(remove_punctuation(each).lstrip(" ").strip("]['"))
        s_MT_Set_PH.add(remove_punctuation(each).lstrip(" ").strip("]['"))
    for w in sw:
        s.add(w)
        s_MT_Set_PH.add(w)
    return s



from gensim.parsing.preprocessing import remove_stopwords
def prepare_MT_words(MT):
    s = set()
    for each in re.split(', | |,',MT):
        each = each.replace('\ ','').lower()
        each = each.replace(' ','')
        each = remove_punctuation(each).lstrip(" ").strip("]['")
        each = remove_stopwords(each)
        if each != '':
            s.add(each)
            s_MT_Set_W.add(each)
    return s

def prepare_criteria_words(df,w):
    l_entities = []
    s = set()
    for i in range(0,df.shape[0]):
        for each in re.split(', | ',df[w][i]):
            each = each.replace('\ ','').lower()
            each = each.replace(' ','')
            each = remove_punctuation(each).lstrip(" ").strip("][')(")
            each = remove_stopwords(each)
            if each != '':
                s.add(each)
        l_entities.append(s.copy())
        s.clear()
    return l_entities

from Levenshtein import setratio as lev

def find_CPC_category_using_similarity(MT,df,col,cpc="",sim=""):
    s_MT = prepare_MT(MT)
    entities = col
    best_similarity=0
    predicted_category=0
    cond = ''
    cri = ''
    for i in range(0,len(entities)):
        if cpc=="" or cpc==df['CPC'][i]:
            if sim == "cos":
                score= cos_similarity(s_MT,entities[i])
            elif sim == "jac":
                score= jaccard_similarity(s_MT,entities[i])
            elif sim == "euc":
                score= euclidean_distance_to_similarity(s_MT,entities[i])
            elif sim == "lev":
                score= lev(list(s_MT),list(entities[i]))
            else:
```

```
                score= cos_similarity(s_MT,entities[i])
            if score > 0.0:
                if score > best_similarity:
                    best_similarity = score
                    predicted_category = df['CPC_category'][i]
                    cond = df['CPC_condition'][i]
                    cri = df['CPC_criteria'][i]
    return predicted_category,cond,cri

def find_CPC_category_using_similarity_words(MT,df,col,cpc="",sim=""):
    s_MT = prepare_MT_words(MT)
    entities = col
    best_similarity=0
    predicted_category=0
    cond = ''
    cri = ''
    for i in range(0,len(entities)):
        if cpc=="" or cpc==df['CPC'][i]:
            if sim == "cos":
                score= cos_similarity(s_MT,entities[i])
            elif sim == "jac":
                score= jaccard_similarity(s_MT,entities[i])
            elif sim == "euc":
                score= euclidean_distance_to_similarity(s_MT,entities[i])
            elif sim == "lev":
                score= lev(list(s_MT),list(entities[i]))
            else:
                score= cos_similarity(s_MT,entities[i])

            if score > 0.0:
                if score > best_similarity:
                    best_similarity = score
                    predicted_category = df['CPC_category'][i]
                    cond = df['CPC'][i]
                    cri = df['CPC_criteria'][i]
    return predicted_category,cond,cri

from collections import Counter

def group_CPC_categories(lst,default=1):
    dict = Counter(lst)
    value = sorted(dict.values(), reverse=True)
    most_ferq = value[0]

    for (key, val) in dict.items():
        if val == most_ferq and int(key)>0:
            return key
        else:
            return default

def compute_accuracy(y_true, y_pred, excluded = 0):
    correct_predictions = 0
    for true, predicted in zip(y_true, y_pred):
        if int(predicted) != 0:
            if true == predicted:
                correct_predictions += 1
            elif isinstance(predicted, str) and true == int(predicted):
                correct_predictions += 1
    accuracy = correct_predictions/(len(y_true)-excluded)
    return accuracy

def compute_accuracy_No_Zeros(y_true, y_pred, excluded = 0):
    correct_predictions = 0
    count = 0
    for true, predicted in zip(y_true, y_pred):
        if int(predicted) != 0:
            if true == predicted:
                correct_predictions += 1
            elif isinstance(predicted, str) and true == int(predicted):
                correct_predictions += 1
            count += 1
    accuracy = correct_predictions/(count-excluded)
    return accuracy

def calculate_excluded(df,df1):
    all_CPCs = set()
    wrong_cat = {}
    wrong_ref = []
    text = ''
    ex = 0
    for i in range(0,df.shape[0]):
        text = df['CPC'][i]+'_'+df['CPC_category'][i]
        all_CPCs.add(text)
        text = ''
    for j in range(0,df1.shape[0]):
        text = df1['CPC'][j]+'_'+df1['Referral_Category'][j]
        if text not in all_CPCs:
            ex+=1
            wrong_ref.append(df1['REF_NUMBER'][j])
            if text not in wrong_cat.keys():
                wrong_cat[text]=1
            else:
                wrong_cat[text]+=1
        text = ''
    return ex,wrong_cat,wrong_ref

def preprocess_CPCs(df,e,c,k):
    #Takes a dataframe, entities column name, and criteria column name
    df['CPC_ent_phr']=prepare_entities_df(df,e)
```

```python
    df['CPC_ent_w']=prepare_entities_df_Words(df,e)
    df['CPC_crit_w']=prepare_criteria_words(df,c)
    df['CPC_kw_phr']=prepare_entities_df(df,k)
    df['CPC_kw_w']=prepare_entities_df_Words(df,k)

def process_similarities(df1,df):
    cate_cos = [[] for i in range(5)]
    cate_jac = [[] for i in range(5)]
    cate_euc = [[] for i in range(5)]
    cate_lev = [[] for i in range(5)]
    aver=[[] for i in range(4)]

    for i in range(df1.shape[0]):
        x,y,z = find_CPC_category_using_similarity_words(df1['Medical_Terms'][i],df,df['CPC_crit_w'],df1['CPC'][i],"cos")
        x1,y1,z1 = find_CPC_category_using_similarity(df1['Medical_Terms'][i],df,df['CPC_ent_phr'],df1['CPC'][i],"cos")
        x2,y2,z2 = find_CPC_category_using_similarity_words(df1['Medical_Terms'][i],df,df['CPC_ent_w'],df1['CPC'][i],"cos")
        x1k,y1k,z1k = find_CPC_category_using_similarity(df1['Medical_Terms'][i],df,df['CPC_kw_phr'],df1['CPC'][i],"cos")
        x2k,y2k,z2k = find_CPC_category_using_similarity_words(df1['Medical_Terms'][i],df,df['CPC_kw_w'],df1['CPC'][i],"cos")
        x3,y3,z3 = find_CPC_category_using_similarity_words(df1['Medical_Terms'][i],df,df['CPC_crit_w'],df1['CPC'][i],"jac")
        x4,y4,z4 = find_CPC_category_using_similarity(df1['Medical_Terms'][i],df,df['CPC_ent_phr'],df1['CPC'][i],"jac")
        x5,y5,z5 = find_CPC_category_using_similarity_words(df1['Medical_Terms'][i],df,df['CPC_ent_w'],df1['CPC'][i],"jac")
        x4k,y4k,z4k = find_CPC_category_using_similarity(df1['Medical_Terms'][i],df,df['CPC_kw_phr'],df1['CPC'][i],"jac")
        x5k,y5k,z5k = find_CPC_category_using_similarity_words(df1['Medical_Terms'][i],df,df['CPC_kw_w'],df1['CPC'][i],"jac")
        x6,y6,z6 = find_CPC_category_using_similarity_words(df1['Medical_Terms'][i],df,df['CPC_crit_w'],df1['CPC'][i],"euc")
        x7,y7,z7 = find_CPC_category_using_similarity(df1['Medical_Terms'][i],df,df['CPC_ent_phr'],df1['CPC'][i],"euc")
        x8,y8,z8 = find_CPC_category_using_similarity_words(df1['Medical_Terms'][i],df,df['CPC_ent_w'],df1['CPC'][i],"euc")
        x7k,y7k,z7k = find_CPC_category_using_similarity(df1['Medical_Terms'][i],df,df['CPC_kw_phr'],df1['CPC'][i],"euc")
        x8k,y8k,z8k = find_CPC_category_using_similarity_words(df1['Medical_Terms'][i],df,df['CPC_kw_w'],df1['CPC'][i],"euc")
        x9,y9,z9 = find_CPC_category_using_similarity_words(df1['Medical_Terms'][i],df,df['CPC_crit_w'],df1['CPC'][i],"lev")
        x10,y10,z10 = find_CPC_category_using_similarity(df1['Medical_Terms'][i],df,df['CPC_ent_phr'],df1['CPC'][i],"lev")
        x11,y11,z11 = find_CPC_category_using_similarity_words(df1['Medical_Terms'][i],df,df['CPC_ent_w'],df1['CPC'][i],"lev")
        x10k,y10k,z10k = find_CPC_category_using_similarity(df1['Medical_Terms'][i],df,df['CPC_kw_phr'],df1['CPC'][i],"lev")
        x11k,y11k,z11k = find_CPC_category_using_similarity_words(df1['Medical_Terms'][i],df,df['CPC_kw_w'],df1['CPC'][i],"lev")


        if x9 != 0:
            default = x9
        elif x5 != 0:
            default = x5
        elif x1 != 0:
            default = x1
        elif x2 != 0:
            default = x2
        elif x3 != 0:
            default = x3
        elif x6 != 0:
            default = x6
        else:
            default = group_CPC_categories([x6,x7,x8])

        cate_cos[0].append(x) if x != 0 else cate_cos[0].append(default)
        cate_cos[1].append(x1) if x1 != 0 else cate_cos[1].append(default)
        cate_cos[2].append(x2) if x2 != 0 else cate_cos[2].append(default)
        cate_cos[3].append(x1k) if x1k != 0 else cate_cos[3].append(default)
        cate_cos[4].append(x2k) if x2k != 0 else cate_cos[4].append(default)
        aver[0].append(group_CPC_categories([x,x1,x2,x1k,x2k],default))
        cate_jac[0].append(x3) if x3 != 0 else cate_jac[0].append(default)
        cate_jac[1].append(x4) if x4 != 0 else cate_jac[1].append(default)
        cate_jac[2].append(x5) if x5 != 0 else cate_jac[2].append(default)
        cate_jac[3].append(x4k) if x4k != 0 else cate_jac[3].append(default)
        cate_jac[4].append(x5k) if x5k != 0 else cate_jac[4].append(default)
        aver[1].append(group_CPC_categories([x3,x4,x5,x4k,x5k]))
        cate_euc[0].append(x6) if x6 != 0 else cate_euc[0].append(default)
        cate_euc[1].append(x7) if x7 != 0 else cate_euc[1].append(default)
        cate_euc[2].append(x8) if x8 != 0 else cate_euc[2].append(default)
        cate_euc[3].append(x7k) if x7k != 0 else cate_euc[3].append(default)
        cate_euc[4].append(x8k) if x8k != 0 else cate_euc[4].append(default)
        aver[2].append(group_CPC_categories([x6,x7,x8,x7k,x8k]))
        cate_lev[0].append(x9) if x9 != 0 else cate_lev[0].append(default)
        cate_lev[1].append(x10) if x10 != 0 else cate_lev[1].append(default)
        cate_lev[2].append(x11) if x11 != 0 else cate_lev[2].append(default)
        cate_lev[3].append(x10k) if x10k != 0 else cate_lev[3].append(default)
        cate_lev[4].append(x11k) if x11k != 0 else cate_lev[4].append(default)
        aver[3].append(group_CPC_categories([x,x1,x2,x1k,x2k,x3,x4,x5,x4k,x5k,x6,x7,x8,x7k,x8k,x9,x10,x11,x10k,x11k]))

    df1['Cat_Crit_cos'] = cate_cos[0]
    df1['Cat_Ent_Ph_cos'] = cate_cos[1]
    df1['Cat_Ent_cos'] = cate_cos[2]
    df1['Cat_kw_Ph_cos'] = cate_cos[3]
    df1['Cat_kw_cos'] = cate_cos[4]
    df1['Cat_Avg_cos'] = aver[0]
    df1['Cat_Crit_jac'] = cate_jac[0]
    df1['Cat_Ent_Ph_jac'] = cate_jac[1]
    df1['Cat_Ent_jac'] = cate_jac[2]
    df1['Cat_kw_Ph_jac'] = cate_jac[3]
    df1['Cat_kw_jac'] = cate_jac[4]
    df1['Cat_Avg_jac'] = aver[1]
    df1['Cat_Crit_euc'] = cate_euc[0]
    df1['Cat_Ent_Ph_euc'] = cate_euc[1]
    df1['Cat_Ent_euc'] = cate_euc[2]
    df1['Cat_kw_Ph_euc'] = cate_euc[3]
    df1['Cat_kw_euc'] = cate_euc[4]
    df1['Cat_Avg_euc'] = aver[2]
    df1['Cat_Crit_lev'] = cate_lev[0]
    df1['Cat_Ent_Ph_lev'] = cate_lev[1]
    df1['Cat_Ent_lev'] = cate_lev[2]
    df1['Cat_kw_Ph_lev'] = cate_lev[3]
    df1['Cat_kw_lev'] = cate_lev[4]
    df1['Cat_Avg'] = aver[3]
```

```python
    df1.replace(to_replace=[None], value=0, inplace=True)


def print_Data():
    print (s_MT_Set_PH)
    print('____________________________________________________________________________________________________')
    print (s_MT_Set_W)

def print_results(df1,df):
#     ex,wrong_cat,wrong_ref = calculate_excluded(df,df1)
    ex=0
    Cat_Crit_cos_m = (df1['Cat_Crit_cos'] == 0).sum()
    Cat_Ent_Ph_cos_m = (df1['Cat_Ent_Ph_cos'] == 0).sum()
    Cat_Ent_cos_m = (df1['Cat_Ent_cos'] == 0).sum()
    Cat_kw_Ph_cos_m = (df1['Cat_kw_Ph_cos'] == 0).sum()
    Cat_kw_cos_m = (df1['Cat_kw_cos'] == 0).sum()
    Cat_Avg_cos_m = min(Cat_Crit_cos_m,Cat_Ent_Ph_cos_m,Cat_Ent_cos_m,Cat_kw_Ph_cos_m,Cat_kw_cos_m)
    Cat_Crit_jac_m = (df1['Cat_Crit_jac'] == 0).sum()
    Cat_Ent_Ph_jac_m = (df1['Cat_Ent_Ph_jac'] == 0).sum()
    Cat_Ent_jac_m = (df1['Cat_Ent_jac'] == 0).sum()
    Cat_kw_Ph_jac_m = (df1['Cat_kw_Ph_jac'] == 0).sum()
    Cat_kw_jac_m = (df1['Cat_kw_jac'] == 0).sum()
    Cat_Avg_jac_m = min(Cat_Crit_jac_m,Cat_Ent_Ph_jac_m,Cat_Ent_jac_m,Cat_kw_Ph_jac_m,Cat_kw_jac_m)
    Cat_Crit_euc_m = (df1['Cat_Crit_euc'] == 0).sum()
    Cat_Ent_Ph_euc_m = (df1['Cat_Ent_Ph_euc'] == 0).sum()
    Cat_Ent_euc_m = (df1['Cat_Ent_euc'] == 0).sum()
    Cat_kw_Ph_euc_m = (df1['Cat_kw_Ph_euc'] == 0).sum()
    Cat_kw_euc_m = (df1['Cat_kw_euc'] == 0).sum()
    Cat_Crit_lev_m = (df1['Cat_Crit_lev'] == 0).sum()
    Cat_Ent_Ph_lev_m = (df1['Cat_Ent_Ph_lev'] == 0).sum()
    Cat_Ent_lev_m = (df1['Cat_Ent_lev'] == 0).sum()
    Cat_kw_Ph_lev_m = (df1['Cat_kw_Ph_lev'] == 0).sum()
    Cat_kw_lev_m = (df1['Cat_kw_lev'] == 0).sum()
    Cat_Avg_euc_m = min(Cat_Crit_euc_m,Cat_Ent_Ph_euc_m,Cat_Ent_euc_m,Cat_kw_Ph_euc_m,Cat_kw_euc_m)
    Cat_Avg_m = min(Cat_Avg_euc_m,Cat_Avg_jac_m,Cat_Avg_cos_m,Cat_kw_Ph_lev_m,Cat_kw_lev_m)




    print('Accuracy  Missing_Predictions  Overall_Accuracy  Comment')
    print('____________________________________________________________________________________________________')
    print("%.3f     %d                    %.3f" %(compute_accuracy_No_Zeros(df1['Referral_Category'],df1['Cat_Crit_cos'],ex),
                                                Cat_Crit_cos_m,
                                                compute_accuracy(df1['Referral_Category'], df1['Cat_Crit_cos'],ex))
          , "           Using CPC Criteria Words and Cosine Similarity")
    print("%.3f     %d                    %.3f" %(compute_accuracy_No_Zeros(df1['Referral_Category'],df1['Cat_Ent_Ph_cos'],ex),
                                                Cat_Ent_Ph_cos_m,
                                                compute_accuracy(df1['Referral_Category'], df1['Cat_Ent_Ph_cos'],ex))
          , "           Using CPC Entities Phrases and Cosine Similarity")
    print("%.3f     %d                    %.3f" %(compute_accuracy_No_Zeros(df1['Referral_Category'],df1['Cat_Ent_cos'],ex),
                                                Cat_Ent_cos_m,
                                                compute_accuracy(df1['Referral_Category'], df1['Cat_Ent_cos'],ex))
          , "           Using CPC Entities Words and Cosine Similarity")
    print("%.3f     %d                    %.3f" %(compute_accuracy_No_Zeros(df1['Referral_Category'],df1['Cat_kw_Ph_cos'],ex),
                                                Cat_kw_Ph_cos_m,
                                                compute_accuracy(df1['Referral_Category'], df1['Cat_kw_Ph_cos'],ex))
          , "           Using CPC Keywords Phrases and Cosine Similarity")
    print("%.3f     %d                    %.3f" %(compute_accuracy_No_Zeros(df1['Referral_Category'],df1['Cat_kw_cos'],ex),
                                                Cat_kw_cos_m,
                                                compute_accuracy(df1['Referral_Category'], df1['Cat_kw_cos'],ex))
          , "           Using CPC Keywords Words and Cosine Similarity")
    print("%.3f     %d                    %.3f" %(compute_accuracy_No_Zeros(df1['Referral_Category'],df1['Cat_Avg_cos'],ex),
                                                Cat_Avg_cos_m,
                                                compute_accuracy(df1['Referral_Category'], df1['Cat_Avg_cos'],ex))
          , "           Average of All Cosine Similarities")
    print()
    print("%.3f     %d                    %.3f" %(compute_accuracy_No_Zeros(df1['Referral_Category'],df1['Cat_Crit_jac'],ex),
                                                Cat_Crit_jac_m,
                                                compute_accuracy(df1['Referral_Category'], df1['Cat_Crit_jac'],ex))
          , "           Using CPC Criteria Words and Jaccard Similarity")
    print("%.3f     %d                    %.3f" %(compute_accuracy_No_Zeros(df1['Referral_Category'],df1['Cat_Ent_Ph_jac'],ex),
                                                Cat_Ent_Ph_jac_m,
                                                compute_accuracy(df1['Referral_Category'], df1['Cat_Ent_Ph_jac'],ex))
          , "           Using CPC Entities Phrases and Jaccard Similarity")
    print("%.3f     %d                    %.3f" %(compute_accuracy_No_Zeros(df1['Referral_Category'],df1['Cat_Ent_jac'],ex),
                                                Cat_Ent_jac_m,
                                                compute_accuracy(df1['Referral_Category'], df1['Cat_Ent_jac'],ex))
          , "           Using CPC Entities Words and Jaccard Similarity")
    print("%.3f     %d                    %.3f" %(compute_accuracy_No_Zeros(df1['Referral_Category'],df1['Cat_kw_Ph_jac'],ex),
                                                Cat_kw_Ph_jac_m,
                                                compute_accuracy(df1['Referral_Category'], df1['Cat_kw_Ph_jac'],ex))
          , "           Using CPC Keywords Phrases and Jaccard Similarity")
    print("%.3f     %d                    %.3f" %(compute_accuracy_No_Zeros(df1['Referral_Category'],df1['Cat_kw_jac'],ex),
                                                Cat_kw_jac_m,
                                                compute_accuracy(df1['Referral_Category'], df1['Cat_kw_jac'],ex))
          , "           Using CPC Keywords Words and Jaccard Similarity")
    print("%.3f     %d                    %.3f" %(compute_accuracy_No_Zeros(df1['Referral_Category'],df1['Cat_Avg_jac'],ex),
                                                Cat_Avg_jac_m,
                                                compute_accuracy(df1['Referral_Category'], df1['Cat_Avg_jac'],ex))
          , "           Average of All Jaccard Similarities")
    print()
    print("%.3f     %d                    %.3f" %(compute_accuracy_No_Zeros(df1['Referral_Category'],df1['Cat_Crit_euc'],ex),
                                                Cat_Crit_euc_m,
                                                compute_accuracy(df1['Referral_Category'], df1['Cat_Crit_euc'],ex))
          , "           Using CPC Criteria Words and Euclidean Similarity")
    print("%.3f     %d                    %.3f" %(compute_accuracy_No_Zeros(df1['Referral_Category'],df1['Cat_Ent_Ph_euc'],ex),
                                                Cat_Ent_Ph_euc_m,
```

```
                                    compute_accuracy(df1['Referral_Category'], df1['Cat_Ent_Ph_euc'],ex))
          , "           Using CPC Entities Phrases and Euclidean Similarity")
    print("%.3f     %d                  %.3f" %(compute_accuracy_No_Zeros(df1['Referral_Category'],df1['Cat_Ent_euc'],ex),
                                    Cat_Ent_euc_m,
                                    compute_accuracy(df1['Referral_Category'], df1['Cat_Ent_euc'],ex))
          , "           Using CPC Entities Words and Euclidean Similarity")
    print("%.3f     %d                  %.3f" %(compute_accuracy_No_Zeros(df1['Referral_Category'],df1['Cat_kw_Ph_euc'],ex),
                                    Cat_kw_Ph_euc_m,
                                    compute_accuracy(df1['Referral_Category'], df1['Cat_kw_Ph_euc'],ex))
          , "           Using CPC Keywords Phrases and Euclidean Similarity")
    print("%.3f     %d                  %.3f" %(compute_accuracy_No_Zeros(df1['Referral_Category'],df1['Cat_kw_euc'],ex),
                                    Cat_kw_euc_m,
                                    compute_accuracy(df1['Referral_Category'], df1['Cat_kw_euc'],ex))
          , "           Using CPC Keywords Words and Euclidean Similarity")
    print("%.3f     %d                  %.3f" %(compute_accuracy_No_Zeros(df1['Referral_Category'],df1['Cat_Avg_euc'],ex),
                                    Cat_Avg_euc_m,
                                    compute_accuracy(df1['Referral_Category'], df1['Cat_Avg_euc'],ex))
          , "           Average of All Euclidean Similarities")
    print()
    print("%.3f     %d                  %.3f" %(compute_accuracy_No_Zeros(df1['Referral_Category'],df1['Cat_Crit_lev'],ex),
                                    Cat_Crit_lev_m,
                                    compute_accuracy(df1['Referral_Category'], df1['Cat_Crit_lev'],ex))
          , "           Using CPC Criteria Words and Levenshtein Similarity")
    print("%.3f     %d                  %.3f" %(compute_accuracy_No_Zeros(df1['Referral_Category'],df1['Cat_Ent_Ph_lev'],ex),
                                    Cat_Ent_Ph_lev_m,
                                    compute_accuracy(df1['Referral_Category'], df1['Cat_Ent_Ph_lev'],ex))
          , "           Using CPC Entities Phrases and Levenshtein Similarity")
    print("%.3f     %d                  %.3f" %(compute_accuracy_No_Zeros(df1['Referral_Category'],df1['Cat_Ent_lev'],ex),
                                    Cat_Ent_lev_m,
                                    compute_accuracy(df1['Referral_Category'], df1['Cat_Ent_lev'],ex))
          , "           Using CPC Entities Words and Levenshtein Similarity")
    print("%.3f     %d                  %.3f" %(compute_accuracy_No_Zeros(df1['Referral_Category'],df1['Cat_kw_Ph_lev'],ex),
                                    Cat_kw_Ph_lev_m,
                                    compute_accuracy(df1['Referral_Category'], df1['Cat_kw_Ph_lev'],ex))
          , "           Using CPC Keywords Phrases and Levenshtein Similarity")
    print("%.3f     %d                  %.3f" %(compute_accuracy_No_Zeros(df1['Referral_Category'],df1['Cat_kw_lev'],ex),
                                    Cat_kw_lev_m,
                                    compute_accuracy(df1['Referral_Category'], df1['Cat_kw_lev'],ex))
          , "           Using CPC Keywords Words and Levenshtein Similarity")
    print()
    print("%.3f     %d                  %.3f" %(compute_accuracy_No_Zeros(df1['Referral_Category'],df1['Cat_Avg'],ex),
                                    Cat_Avg_m,
                                    compute_accuracy(df1['Referral_Category'], df1['Cat_Avg'],ex))
          , "           Average of All Similarities")



# # Predicting CPCs

# In[14]:



def most_frequent(List):
    return max(set(List), key = List.count)




def Predict_CPC(MT,df,ref):
    s_MT_w = prepare_MT_words(MT)
    s_MT_p = prepare_MT(MT)
    ent = ['CPC_ent_phr','CPC_ent_w','CPC_crit_w','CPC_kw_phr','CPC_kw_w']
    best_similarity=[[0,0,0,0],[0,0,0,0],[0,0,0,0],[0,0,0,0],[0,0,0,0]]
    predicted_CPCs=['','','','','']
    predicted_CPC_ind = 100
    count = 0
    for j in range(5):
        entities = df[ent[j]]
        s_MT = (s_MT_p if (j==0 or j==3) else s_MT_w)
        for i in range(len(entities)):
            improve = 0.0
            cos_score= cos_similarity(s_MT,entities[i])
            jac_score= jaccard_similarity(s_MT,entities[i])
            euc_score= euclidean_distance_to_similarity(s_MT,entities[i])
            lev_score= lev(list(s_MT),list(entities[i]))

            improve += (0.9 if cos_score > best_similarity[j][0] else 0.0)
            improve += (1.1 if jac_score > best_similarity[j][1] else 0.0)
            improve += (0.9 if euc_score > best_similarity[j][2] else 0.0)
            improve += (1.1 if lev_score > best_similarity[j][3] else 0.0)
            if improve > 2.0 :
                best_similarity[j][0] = cos_score
                best_similarity[j][1] = jac_score
                best_similarity[j][2] = euc_score
                best_similarity[j][3] = lev_score
                predicted_CPCs[j] = df['CPC'][i]
    predicted_CPCs = [x for x in predicted_CPCs if x]
    if len(predicted_CPCs) == 0 and ref=='adult':
        predicted_CPCs.append('dysphagia (adult)')
    elif len(predicted_CPCs) == 0 and ref=='paed':
        predicted_CPCs.append('dysphonia/hoarseness (paediatric)')
    if len(list(dict.fromkeys(predicted_CPCs)))<4:
        predicted_CPC = most_frequent(predicted_CPCs)
    else:
        predicted_CPC = most_frequent(predicted_CPCs)
    return predicted_CPC
```

```
# In[15]:


adult_CPC.head()


# In[16]:


adult_CPC = adult_CPC.reset_index(drop=True)
paed_CPC = paed_CPC.reset_index(drop=True)
preprocess_CPCs(df_CPC,'CPC_entities','CPC_criteria','CPC_keywords')
preprocess_CPCs(adult_CPC,'Combined_CPC_entities','Combined_CPC_criteria','Combined_CPC_keywords')
preprocess_CPCs(paed_CPC,'Combined_CPC_entities','Combined_CPC_criteria','Combined_CPC_keywords')


# In[17]:


adult_CPC.head()


# In[18]:


Predict_Adult_CPC=Predict_Adult_CPC.reset_index(drop=True)
Predict_Paed_CPC=Predict_Paed_CPC.reset_index(drop=True)

for i,each in enumerate(Predict_Adult_CPC['Medical_Terms']):
    Predict_Adult_CPC['CPC'][i] = Predict_CPC(each,adult_CPC,'adult')

for i,each in enumerate(Predict_Paed_CPC['Medical_Terms']):
    Predict_Paed_CPC['CPC'][i] = Predict_CPC(each,paed_CPC,'paed')

Predict_Paed_CPC.head()


# In[19]:


merged_df = pd.concat([Predict_Paed_CPC, Predict_Adult_CPC,available_CPC])


# In[20]:


merged_df.describe()


# In[21]:


merged_df.head(100)


# # Predicting Category Using Phase I Code On Predicted and Available CPCs

# In[22]:


merged_df=merged_df.reset_index(drop=True)
df_CPC=df_CPC.reset_index(drop=True)
process_similarities(merged_df,df_CPC)


# In[23]:


merged_df.head()
```

Medical Terms Sample

{'ct skeletal surveys', 'ood', 'earphones ox', 'vocal cord paresis',
'extensive dental disease', 'systolic excursion', 'suctioned out', '
vertigo occasionally', 'appetite has improved', 'darriers disease',
'middle ear mobility', 'embolus', 'lowpitched', 'lesione', 'feels
thts', 'retinal artery thrombosis', 'parforation', 'ultrasound
guidance', 'noe', 'slow wave sleep', 'suctions', 'sosinophilia',
'she', 'hydrocortisone acetate', 'gastrooesophageal reflux disease',
'fill the effected ear canal', 'radical prostatectomy', 'felt worse',
'glycerol ear drops', 'intracerebral haemorrh', 'fats', 'magazine',
'small turbinates', 'feel nodes', 'polyps sinusses', 'administrative
observations', 'sleep disordred breathing', 'right maxillary sinus',
'dairy', 'dymesta', 'aliniaal', 'frontal resurfacing',
'hypotension14', 'cyanotic episode', 'speech good', 'concave
depression', 'vacuum extraction', 'breathing rhythm', 'vocal chord
nerve injury', 'bright light', 'active liver disease', 'open
referral', 'scc of throat', 'atrial ectopic', 'headcahes',
'maligna15', 'gival proumowns', 'ulceration lesion', 'enlarged
obstructive tonsil', 'minocycline', 'conversational routines',
'grometts', 'blodo', 'ear canal lesion', 'sensorineural hearingloss',
'abrasion', 'eformotero', 'neoplastic naturo', 'enlarged tonslls',
'reacts normally', 'sturred', 'run down', 'wakes refreshed',
'appearing tired', 'oesophageal dilation', 'tonsillar enlargment',
'infeciton', 'hav', 'align normally', 'failry', 'otitis extema',
'diseaseinthefloorofthesphenoid', 'lispro', 'sorc dioscharging',
'weight is steady', 'cscope', 'ic dysfunction', 'ethmoidal mucosal
disease', 'focal mucosal thickening', 'syringe1', 'swallow
dysfunction', 'dyshpidaema', 'benzoy', 'cranial suture closure',
'ionised calcium', 'nutritional gain', 'gbs bacteraemia', 'normal
echotexture', 'liquid', 'capd', 'vascular lump', 'nasonex spray',
'ward of collection', 'liver recheck', 'disturbing', 'new referral',
'looking blockage', 'ear suctions', 'conspicuous nucleoli', 'ubescent
deficiency', 'nasal spur', 'osteophytes', 'difficult hyperactive
behaviours', 'symptorns', 'hyperkeratosis', 'tongue carcinoma',
'ribosomal', 'susceptible', 'allergic hypertrophy', '2018behaviour
disorder', 'solid body', 'rabepraz', 'procedurea', 'mucosal
prolapse', 'esrcrp', 'dleur', 'tensive rhinosinusitis', 'thyroid
tenderness', 'hypersomnolence', 'adenoids enlarged', 'sialolith',
'parotid lasion', 'hypog', 'lytic bone lesions', 'polypoidal
changes', 'ulcerative oesophagitis', 'radiofrequency ablation',
'infanrix hexa', 'mastoid sinus opacification', 'sleep maintenance
issues', 'staph', 'monitoring growth', 'brain lesions', 'breathing
artifact', 'chemical cauterisation', 'allow', 'adenitis', 'lantus
solostar pen', 'normal contour', 'multiple', 'surface disruption',
'mirtazapine', 'calf2012diabetes mellitus', 'ocs', 'discoordinate',
'admini', 'laminectomyrbwh', 'closed nasal bone fracture', 'coronary
irregularities', 'mass effect', 'cipa', 'hearing was reduced',
'tender tonsillar lymph nodes', 'versus', 'involve', 'tm intact',
'minced moist diet', 'largest', 'antihypertensive agents',
'motorvehicle accidents', 'fussy eater', 'masticaton', 'metastatic
lymph node', 'leucs', 'aiello', 'dateconditionpulmonary valve
stenosis', 'fds ptale', 'reduced sense of smell', 'blocked fooling',
'ct guided injections', 'nandyn', 'elevated resistive indices',
'oppositional defiant disorder', 'needing po', 'ulcers', 'mas',
'increased sclerosis', 'perivascular', 'saccade or tracking',
'sensation of something stuck in her throat', 'embrane perforation',
'back pain25', 'diseaseanxiety2019endoscopy', 'atrovent forte',
'satisfactory cochlea function', 'clear his throat', 'mastoid areas',

'bit', 'flag', 'choking in his sleep', 'well aferile', 'intelligent', 'pus like discharge', 'fenac', 'articulation defect', 'episodic dizziness', 'psoriasisesomeprazole', 'osteophyteatc4', 'inattentive hyperactive disorder', 'antiinflammatory drugs', 'cholesteatome', 'plosion on each sound', 'dysphag', 'papular lesion', 'glaucomagestational diabetes mellitusburning feet syndrome25', 'irin', 'frenctomy', 'clonidine hydrochloride', 'alergeis', 'allerigic rhinitis', 'nasal resitance', 'vascular appearance', 'sensory processing difficulties', 'pituitary bright spot', 'sturtor', 'low magnesium', 'impacting his speech', 'delay', 'smores', 'nasal cavity fullness', 'whiteyellow', 'biceps reflex', 'tramadol', 'loneliness', 'elling', 'no surface collection', 'wondered about his turbinates', 'grommet placement', 'parotid swelling', 'airway distress', 'voice is back to normal', 'reflux pain', 'healthy lifestyle modifications', 'imaging', 'sleep is broken', 'oripro', 'aricept', 'dificit', 'nas', 'squamous cell metastatic disease', 'tocacorten', 'skin flap', 'cono', 'fexofenadine', 'off balance', 'vildagliptin', 'bone erosion', 'looked very well', 'susceptibility tasting', 'siderosis', 'selenium', 'perioral dermatitis', 'central ulceration', 'matter changes', 'insulin lispro', 'thickened cortices', 'dyr', 'subaortic', 'frequencies', 'systemic autoimmune disease', 'es', 'asd traits', 'summary asthma', 'dystonia', 'trouble both falling asleep', 'tonsillitis chronically enlarged', 'ultrasoun', 'montelukast insomnia', 'appetite slowly improving', 'obstructed frontal recesses', 'elevation in crf', 'dacryocystorhinostomy', 'appendectomy', 'trouble with nose', 'small infarcts', 'liproscing excellent', 'maslp', 'signifocant', 'covid 19 situation', 'nicotine replacement', 'sxt', 'nasal inspection', 'extraaxial hemorrhage', 'normal cervical spine', 'picato', 'nodal volumes', 'paracetamolcodeine g phosphate', 'salmeterol xinafoate', 'thoracic curvature', 'coccal skin infections', 'renecol', 'coronary angioplasty with stent', 'episodic vertigo', 'zactin', 'focal haematoma', 'feel panicky', 'hypoglycacmia', 'shus', 'maligrancy', 'endone', 'calcium trihydrate', 'blocked tear', 'maxillary sinus hyperplasia', 'atenolol',…
