# Supplementary material for: Artificial intelligence in medical referrals triage based on Clinical Prioritization Criteria
Source: Front Digit Health. 2023 Oct 27;5:1192975. doi: 10.3389/fdgth.2023.1192975 (PMC10642163; doi:10.3389/fdgth.2023.1192975)
Supplement: Supplementary file 2 [file Datasheet2.pdf]

Supplementary File 2

Hyperparameters values: We use default values for any hyperparameter no listed in the table below.

Algorithm	Hyperparameter	Range	Optimised Value
Stochastic gradient descent (SGD) (sklearn.linear_model.SGDClassifier)			
	Loss	['hinge', 'log_loss', ' perceptron']	perceptron
	penalty	['l1', 'l2', 'elasticnet']	L2
	alpha	[0.0001, 0.001, 0.01, 0.1, 1, 10, 100, 1000]	0.0001
	max_iter	[100, 1000, 5000]	1000
	learning_rate	['constant', 'optimal', 'invscaling', 'adaptive']	Optimal
	eta0	[0, 1, 10, 100]	1
Random forest (sklearn.ensemble.RandomForestClassifier)			
	n_estimators	[10, 50, 100, 200, 400, 1000]	400
	criterion	['gini', 'entropy', 'log_loss']	gini
	max_depth	[3, 5, 7, 9, 11, 13, 15]	7
	min_samples_split	[5, 10, 20, 50]	10
	min_samples_leaf	[1, 2, 3, 4, 5]	4
	max_features	['auto', 'sqrt']	auto
	bootstrap	[True, False]	True
Gradient boosting (sklearn.ensemble.GradientBoostingClassifier)			
	loss	['log_loss', 'exponential']	exponential
	learning_rate	[0.001, 0.005, 0.01, 0.1]	0.005
	n_estimators	[10, 100, 500, 1000]	100
	subsample	[0.4, 0.6, 0.8, 1.0]	0.6
	min_samples_split	[5, 10, 50]	50
	min_samples_leaf	[1, 3, 5]	5
	max_depth	[3, 9, 15]	9
	max_features	[3, 7, 11, 15]	7
Multilayer perceptron (sklearn.neural_network.MLPClassifier)			
	hidden_layer_sizes	[(10,), (20,), (30,)]	(20,)
	activation	['tanh', 'relu']	relu
	solver	['lbfgs', 'sgd', 'adam']	adam
	alpha	[0.0001, 0.001, 0.01, 0.05, 0.1]	0.0001
	learning_rate	['constant', 'adaptive']	constant
light gradient boosting machine (light GBM) (lightgbm.LGBMClassifier)			
	num_leaves	[30, 50, 70, 90, 100]	90
	max_depth	[3, 5, 7]	25
	learning_rate	[0.001, 0.005, 0.01, 0.1]	0.001
	n_estimators	[10, 100, 500, 1000]	100
	subsample	[0.4, 0.6, 0.8, 1.0]	1.0
	colsample_bytree	[0.1, 0.3, 0.5, 0.7, 1.0]	0.5
	reg_alpha	[0.1, 0, 1, 5, 10, 100]	1
	reg_lambda	[0.1, 0, 1, 5, 10, 100]	5

Extreme gradient boosting (Xgboost) (xgboost.XGBClassifier)		
max_depth	[3, 5, 7]	7
eta	[0.001, 0.005, 0.01, 0.1]	0.001
n_estimators	[10, 100, 500, 1000]	100
subsample	[0.5, 0.7, 1]	1
colsample_bytree	[0.1, 0.3, 0.5, 0.7, 1.0]	0.1
alpha	[0.1, 0, 1, 5, 10, 100]	10
lambda	[0.1, 0, 1, 5, 10, 100]	10
